# Evaluation of a Probe-Based PCR-ELISA System for Simultaneous Semi Quantitative Detection and Genotyping of Human Cytomegalovirus (HCMV) Infection in Clinical Specimens

**DOI:** 10.2174/1874285801711010083

**Published:** 2017-05-31

**Authors:** Majid Talkhabifard, Naeme Javid, Abdolvahab Moradi, Amir Ghaemi, Alijan Tabarraei

**Affiliations:** 1 Faculty of Advanced Medical Technologies, Golestan University of Medical Sciences, Gorgan, Iran; 2 Department of Microbiology, Golestan University of Medical Sciences, Gorgan, Iran; 3 Infectious Diseases Research Center, Golestan University of Medical Sciences, Gorgan, Iran

**Keywords:** PCR-ELISA, Human cytomegalovirus, Glycoprotein B, Semi-quantitative detection, Genotyping

## Abstract

**Background:**

Human cytomegalovirus (HCMV) is a common opportunistic pathogen that causes serious complications in immunosuppressed patients and infected newborns. In this study, PCR-ELISA was optimized for semi-quantitative detection of infection in clinical specimens and simultaneous genotyping of glycoprotein B for 4 major genotypes, due to its significance.

**Method:**

During DIG-labeling PCR, a pair of primers amplifies a fragment of variable region of the glycoprotein B encoding sequence. Under optimized conditions, labeled Target amplicons hybridize to biotinated specific probes and are detected in an ELISA system.

**Results:**

PCR-ELISA system showed specific performance with detection limit of approximately 100 copies of CMV DNA. The linear correlation was observed between the PCR-ELISA results (OD) and logarithmic scale of CMV (r=0.979). Repeatability of PCR-ELISA detection system for intra-assay and inter-assay was evaluated for negative and positive samples. In optimized conditions of hybridization, differentiation between genotypes of glycoprotein B was feasible using genotype-specific probes in PCR-ELISA genotyping system.

In comparison with sequencing method, genotyping system was confirmed with kappa index of 1.

**Conclusion:**

PCR-ELISA is proposed as an applicable and reliable technique for semi-quantitative diagnosis and typing of the infection. This technique is flexible to apply in a variety of molecular fields.

## 1 INTRODUCTION

Human cytomegalovirus is an opportunistic infection with global prevalence that can act as a dangerous pathogen in immunocompromised persons. Especially, transplant recipients, patients with acquired immune deficiency syndrome and congenital infected newborns are at risk of developing CMV disease [1]. This virus is most important cause of congenital viral infection that commonly leads to serious complications including encephalitis, chorioretinitis, pneumonia, hearing loss, microcephaly and mental retardation. Accurate early detection and quantification of CMV infection in clinical specimens is necessary for prognosis and control of the CMV disease in high-risk patients. This is especially important in newborns with congenital CMV infection, because of serious irreparable clinical complications of CMV disease in this group [2-4].

CMV glycoprotein B (gB) is the major envelope component of CMV, and is encoded by highly variable gene UL55. Glycoprotein B plays an important role in viral entry, cell-to-cell transmission and mediates fusion of infected cells for establishment and spread of the infection [5, 6]. Also, glycoprotein B is an important immunogenic target for the host immune system, as it is majorly responsible for induction of neutralizing antibodies in the natural host [7, 8].

Based on variable region of the glycoprotein B that mainly encompasses the protease cleavage site, it is classified into four major genotypes that exist among different strains of HCMV [9, 10].

Correlation Possibility between the glycoprotein B genotypes and occurrence of CMV disease in susceptible people have been the target of many studies [11, 12]. Also gB is regarded as an interesting target for preventive CMV vaccine production [13].

PCR-ELISA (Polymerase Chain Reaction-Enzyme Linked Immuno Sorbent Assay) is a diagnostic procedure, involving coupling of PCR and ELISA techniques. By this system, the desired target sequence of amplified DNA product of PCR for Sample as a template is detected by its specific oligonucleotide probe, and this hybrid becomes visible due to enzymatic activity in ELISA system [14, 15]. As well as using the standard curve, it is possible to quantify the results [15, 16].

Due to the high flexibility of this method for selection and optimization of various probes, and considering high diagnostic specificity generated by specific primers and probes, this method is suitable for diagnosis and screening of different types of pathogens and microorganisms [14, 15, 17]. Also in the optimum conditions, this method is able to identify and differentiate various mutations and genotypes as well as mixed genotypes [14, 15, 18].

In the present study, PCR-ELISA system was established for semi-quantitative detection of the CMV infection in clinical samples, and used as a method to study glycoprotein B for various genotypes.

## 2 MATERIALS AND METHOD

### 2.1 Primers and Probes

Previously published nucleotide sequences of the CMV gB gene (*UL55*), including gB1, gB2, gB3, and gB4 genotypes and their variants were obtained from GenBank [NCBI]. Sequences were aligned using generunner software to distinguish between homologous and heterogeneous sequence regions (Accession number: GU365817-GU365824) [19].

Specific oligonucleotide primers and probes were designed based on highly variable proteolytic cleavage site region of the gB gene (between amino acids 459 and 460, HCMV strain AD169).

Primers were designed on highly conserved regions for described four genotypes (between nucleotides 1259 and 1485 of gB gene according to the HCMV strain AD169 sequence, Gene Bank accession number X17403) to amplify a segment of variable region (gB1 and gB4, 229-bp; gB2, 226-bp; gB3, 223-bp).

Subsequently, sequences of the primer pairs were evaluated by NCBI nucleotide BLAST to confirm specificity of the primers.

For the region of interest, a consensus probe was selected to detect the CMV gene, *via* binding with same specificity to all four genotypes of the gB. As well as four unique genotype-specific probes were designed to differentiate between the four gB genotypes.

Designed genotyping probes are complementary to each of the genotypes of interest, while they have lowest cross reaction to non-proprietary sequences. Formation probability of secondary structures was evaluated in temperature conditions of the experiments.

All oligonucleotides were designed using Generunner and BLAST (NCBI) programs.

Probes were 5’-labeled by biotin for immobilizing the hybrid of target sequence with the probe, in the ELISA DIG detection stage, by the manufacturer.

### 2.2 Clinical Specimens and Ccontrols

Positive controls were prepared in the form of plasmid (produced by Bioneer company) for the interest region of four gB genotypes (according to described sequences) to set up and optimize the PCR-ELISA.

HCMV-positive urine samples of 16 infants that had previously been isolated by molecular methods were used as clinical controls for PCR-ELISA system.

Urine samples were DNA extracted using boiling method according to previously published protocol [20].

### 2.3 PCR Conditions

5 microliter of extracted DNA from urine samples were used as the template for digoxigenin labeling PCR, as well as the positive and negative controls in final volume of 25 µl containing 1X PCR Buffer (100 mM Tris-HCl, pH 8.3, 500 mM KCl) (Roche); 1.5 U of Taq DNA Polymerase (Roche); 0.4 µM of each primer; 3 mM MgCl2 and 0.2 mM of each dATP, dCTP, dGTP, 0.19 mM dTTP, 0.01 mM digoxigenin-11-dUTP (Roche).

The thermocycling programs, preceded by 5 min at 95°C to initial denaturing the sample DNA, were followed by 35 cycles of 1 min at 95°C, 1 min at 61°C and 30 s at 72°C. Finally, these were followed by, 5 min at 72°C for final elongation.

0.5 ng of each gB control plasmids were used as positive controls template for PCR setup and optimization. Also negative controls containing all of the reaction components except DNA were used for contamination surveillance.

To reduce the possibility of contamination, material preparation room was separated from sampling and amplification place and also sterile, disposable laboratory supplies were used.

### 2.4 Detection of PCR Products (HCMV Diagnosis)

After thermocycling, the detection of the DIG-labeled PCR products was carried out using the PCR ELISA, DIG Detection Kit (Roche), according to the manufacturer’s recommendations.

For the CMV detection PCR-ELISA system, 2.5 µl of DIG-labeled PCR product was mixed with 10 µl of denaturation solution in a sterile micro tube and incubated for 10 min at room temperature (10 µl denaturation solution added to a separate empty micro tube for reagent blank), then hybridization solution containing 20 pmol/ml of the consensus biotin 5’-labeled probe was added to a final volume of 125 µl (initially, for optimize procedure, ،different concentrations of the probe were tested to get desirable results). 100 µl from each micro tube transferred rapidly to streptavidin-coated microplates and was incubated in hybridization oven at 37°C for 1 h with shaking (120 rpm), and then microplates were washed four times with washing solution. 100 µl of anti-digoxigenin peroxidase conjugate working solution (each mU of anti-DIG POD diluted 100-fold in conjugate dilution buffer for working solution) was added to each microplate and was incubated for 30 min at 37°C with shaking. Microplates were washed four times again, then 100 µl of ABTS solution was added as substrate to each reaction microplate and was incubated in the dark for 30 min at 37°C with shaking. Finally, absorbance of each well was read at 405 nm (reference wavelength 492 nm) using an ELISA plate reader.

DIG-labeled PCR products of the gB control plasmids (Primary concentration of 0.5 ng of controls) were used for optimization of the PCR-ELISA. Each HCMV detection PCR-ELISA reaction was carried out with CMV positive sample [clinical urine sample] and CMV negative sample as controls. For semi-quantitative assessment of amplified HCMV, results of HCMV detection PCR-ELISA were quantified from 0.5+ (weakly positive) to 4+ (strongly positive). All results represent mean values of triplicate experiments.

Mean absorbance value of the negative controls (20 samples of HCMV-negative urine) plus three standard deviations was determined as cut-off value for CMV detection PCR-ELISA (0.067+(3×0.015)=0.112) [14].

Samples with absorbance greater than 0.112 were considered as positive.

#### 2.4.1 Sensitivity and Specificity of HCMV Detection PCR-ELISA

For sensitivity assessment of the optimized HCMV detection PCR-ELISA, 10-fold serial dilutions with an initial value of 35 pg of plasmid Containing gB2 positive control (approximately 10^7^ copy number) were prepared as PCR-ELISA template using consensus probe as well as five microliters of the PCR products were electrophoresed on 2% agarose gel and stained by ethidium bromide for further study.

To evaluate the specificity of the Launched PCR-ELISA for detection of HCMV genome, tests were performed on extracted DNA of HCMV-negative human serum, a clinical specimen positive for HSV while HCMV negative, and a HCMV-negative urine sample.

### 2.5 gB Genotyping

Designed genotype-specific probes were used to discriminate four gB genotypes of each other in PCR-ELISA genotyping system.

For each of the DIG-labeled PCR products of CMV positive samples, four separate DIG-detection tests were performed, similar to the procedures described above, with each of the genotype-specific probes.

For proper differentiation of the genotypes and minimizing of non-specific bindings, optimum incubation temperature for hybridization step was determined at 55°C.

Under these conditions and 20 p mol/ml concentrations of the gB1, gB2, gB4 biotin-labeled specific probes and 40 p mol/ml of the gB3 biotin-labeled specific probe for hybridization, satisfactory results were obtained and genotyping became feasible.

To determine sensitivity of the genotyping system and draw genotyping pattern, PCR-ELISA genotyping was performed with different concentrations of gB control plasmids as template.

Labeled PCR products of 16 HCMV-positive urine samples initially were analyzed semi-quantitatively in DIG detection ELISA through consensus probe as described above, then genotypes of labeled PCR products were evaluated using genotype specific probes in genotyping ELISA system according to the achieved pattern.

#### 2.5.1 Verification of the Genotyping System

To verify the results of the genotyping PCR-ELISA, the amplified products of described 16 clinical specimens were sequenced in desired region and results were compared with the obtained results of the launched genotyping PCR-ELISA. To reduce amplification errors, PCR was performed using Pfu polymerase enzyme instead of Taq polymerase for sequencing.

### 2.6 Statistical Analysis

SPSS statistical software [version 16.0] and excel program (Microsoft office 2007) were used for analysis of the data. The kappa index was calculated to determine the measure of agreement for genotyping system. Also the absorbance values and concentration of the control plasmids in the form of logarithmic scale were analyzed by Pearson's correlation coefficient.

To evaluate repeatability of the CMV detection PCR-ELISA system, coefficients of variation of intra-assay and inter-assay were calculated for absorbance value (S.D./mean X 100%).

## 3 RESULTS

### 3.1 CMV Detection PCR-ELISA System Development

To assess the sensitivity of the CMV detection PCR-ELISA system, according to the Cut-off value, obtained results of different concentrations of the gB control were shown a limit of less than 0.35 femtogram (Optical density = 0.22) of the plasmid (less than 100 copies) for the detection of the virus genome, that was approximately equal to the results of the electrophoresis of the amplified products on 2% agarose gel and stained with ethidium bromide.

**Fig. (1):**
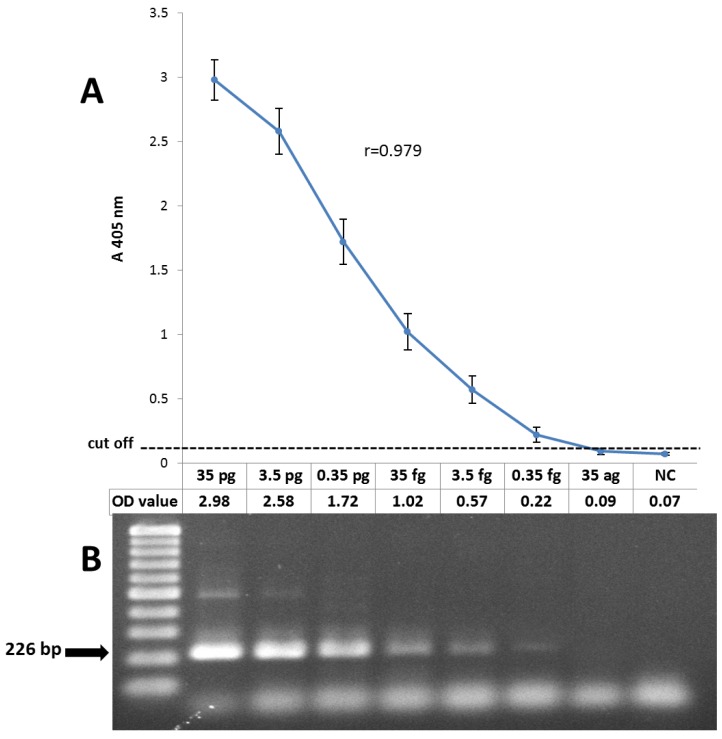
**Analytical sensitivity of CMV detection PCR-ELISA system**. **A.** The results of the PCR-ELISA for 10-fold serial dilution of control plasmid containing gB2 sequence. The detection limit was 0.35 fg of plasmid DNA (~ 100 copies). The Pearson’s correlation coefficient between PCR-ELISA OD and logarithmic scale of plasmid DNA was 0.979 that represents the linear regression. **B.** Electrophoresis of the PCR products by 2% agarose gel with ethidium bromide staining (226 bp).

The correlation between the absorbance value of the PCR-ELISA and concentration of the DNA template (logarithmic scale of gB2 control plasmid) were analyzed and Pearson's correlation coefficient of 0.979 was calculated (Fig. **1**).

To evaluate the specificity of the HCMV detection system, obtained results of performing the HCMV detection PCR-ELISA on specimens of HCMV- negative human serum and urine samples, and a clinical case that was HSV1- positive and HCMV-negative, indicated the insignificant nonspecific reactions (optical density lower than the cutoff value) in this detection system Fig. (**2**).

To calculate the intra-assay coefficients of variation for absorbance value in CMV detection PCR-ELISA system, 2 negative and 2 positive urine samples were examined in four tests individually in a run. Intra-assay CVs for negative samples were 6.3% and 8.2%; the CVs for the positive samples were 1.6% and 2.5%. As well as to measure the inter-assay CV, 2 negative and 2 positive urine samples were assessed in four separate experiments on four different days. Inter-assay CVs were 15.2% and 18.5% for negative samples, and variations were 7.3% and 12.1% for positive samples.

### 3.2 CMV gB Genotyping PCR-ELISA System Development

To achieve genotyping pattern for each of the four genotypes, under optimized conditions, PCR-ELISA genotyping system was carried out with several different concentrations of control samples as template. Using the obtained results, the graphs were plotted as pattern of genotyping Fig. (**3**). Using these models, it is possible to distinguish the genotypes and mix genotypes in samples.

### 3.3 Analysis of Clinical Samples

Discussed HCMV-positive urine specimens were initially assessed by HCMV detection PCR-ELISA semi-quantitatively by using consensus probe and CMV genome was detected in all 16 clinical samples. Next, types of gB were determined by using specific probes in the genotyping system. Every four genotypes were found in the samples, but no mixed infection of two or more genotypes was detected by the system.

To evaluate the accur acy of genotyping system, measure of agreement was assessed between results of the sequencing (as the reference method) and the genotyping system. Method was confirmed according to the calculated kappa index of 1.

## DISCUSSION

Due to the complications of the CMV disease, improvement of molecular methods for study and proper diagnosis of infections in clinical samples seems necessary. In this study, PCR-ELISA system was launched as a semi-quantitative method for detection of CMV infections and differentiation of four major genotypes of the gB in clinical specimens.

The optimized CMV detection PCR-ELISA is able to detect less than 100 copies of target DNA per reaction in specimens; this diagnostic sensitivity is comparable with the results of the previous studies (between 10 to 200 copies of the target gene) that were performed for the diagnosis of CMV infection [21-23].

To determine the cutoff value, various studies have used several formulas that are mostly based on similar principles (mean absorbance value of the negative controls + 1 to 3 times of standard deviation) [14, 22]. In this study, mean absorbance value of the negative controls + 3 SD was used for cut off value to reduce false positives and increase the specificity.

The sensitivity comparison of this method with the results obtained by electrophoresis of PCR products on agarose gel and stained by ethidium bromide, showed no significant difference between the two methods, which was similar to some previous studies [21, 24].

A significant correlation was shown between concentrations of the template DNA in logarithmic scale [10^2^-10^7^ copies of the gB2 control plasmid] and the results (optical density) of the CMV-detection PCR-ELISA with Pearson's correlation coefficient of 0.979 that potentially enables the diagnostic system to quantify detection of viral genome.

Assay method in the dig detection stage is end point colorimetric reaction (enzyme-substrate reaction), therefore in this study, was considered as a semi-quantitative assay.

According to previous studies, viral load can be involved in prognosis and severity of the disease. For this reason, quantitative measurements can help to control the course of infection [25-27].

There were negligible nonspecific reactions (Below Cutoff) with the human genome and HSV1 that indicated a good specificity of system for detection of CMV in clinical samples, using employed oligomers.

For typing the described gene, at specified hybridization temperature, reduced non-specific bindings and differentiation between genotypes were possible.

The results of the genotyping were confirmed by sequencing, however, in the case of mixed infections, sequencing method would not be able to distinguish it.

Commonly used methods for detecting the CMV infection in clinical samples are pp65 antigenemia assays, conventional qualitative PCR and Real-time PCR.

According to previous studies, using PCR-based methods, CMV infection can be recognized at least a week earlier than pp65 antigenemia assay in blood specimen of people at risk [28-30]. It is shown that diagnosis of infection in the early stages of the disease can be helpful to start preemptive antiviral therapy and management of infection in these groups [31]. Unfortunately, latent CMV in healthy individuals can also be detected by PCR that is clinically insignificant infection. To overcome this limitation, Quantification of PCR can be useful to monitor CMV replication and differentiate active infection from inactive [30, 31].

On the other hand, pp65 antigenemia assays are quantifiable through the identification of infected white blood cells. But for the direct detection of the virus in plasma, serum, urine, saliva and other secretions, viral particles are mostly in the form of extracellular, pp65 antigenemia assay; however unlike PCR-based method, it is not a suitable approach. Even satisfactory results may not be achieved with this method for blood samples from patients with leukopenia [28-30].

Because of the high flexibility of PCR-ELISA to selecting and applying the various probes, this technique is able to optimize a diverse range of fields [14, 15]. Hence, this method can be an alternative for Real-time PCR method with comparable specificity but lower diagnostic sensitivity [15].

This method by conventional equipment, without using hazardous materials, is able to carry out work during day in typical laboratories. 96 reactions ELISA plates with simple procedure make it possible to perform a large number of tests in each round of work that makes it an appropriate economical method for screening and epidemiological approaches.

Due to the little volume of the amplified products, it is required to perform ELISA, therefore several Dig-detection ELISA tests can be carried out on each reaction product. Because of its open systems, the procedure of Dig-detection ELISA is highly flexible, so that for different purposes, probes can be substituted, deleted or added. ELISA system can be performed using different optimized probes with different settings simultaneously in a round work on a single reaction of the labeled PCR products.

No automated system and possibility of contamination due to open system, high duration of the procedure (6-8 hours) and semi quantified results are some of the disadvantages of PCR-ELISA in comparison with Real-time PCR. This flexibility of the procedure reduces the costs and increases the speed and convenience for performing the test.

## CONCLUSION

PCR-ELISA could be proposed as a useful technique for diagnosis and management of infections that are clinically valuable to measure quantitatively.

## Figures and Tables

**Fig. (2):**
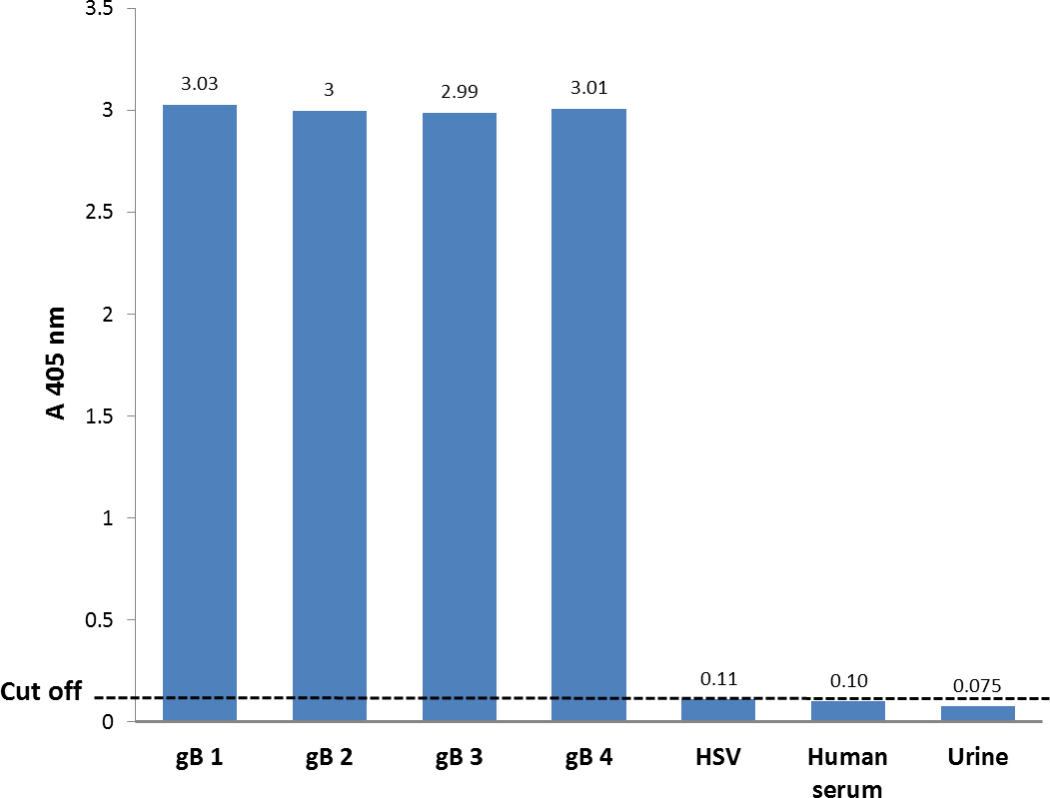
**Analytical specificity of CMV detection PCR-ELISA system**. Comparison the results of the CMV detection PCR-ELISA for specimens of; HCMV- negative human serum, urine samples, and HSV1- positive sample, and also 0.5 ng of each of the gB control plasmids, based on cut off value.

**Fig. (3):**
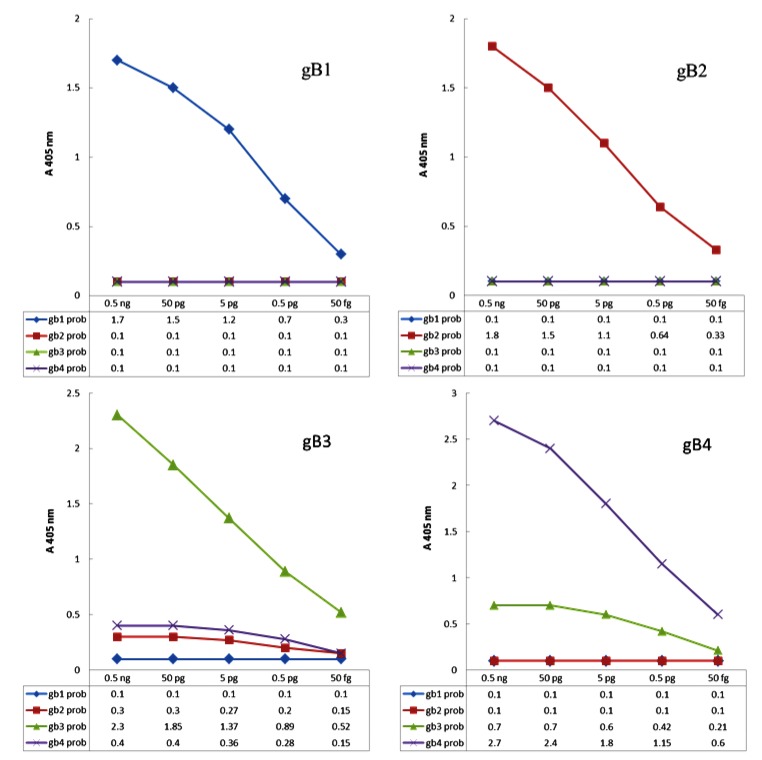
**Genotyping models**; Resulting from 10-fold serial dilutions of four gB types of control plasmids performed by genotype-specific probes in the genotyping system at hybridization temperature of 55°C.
